# Multimodal Morphometric Similarity Network Analysis of Autism Spectrum Disorder

**DOI:** 10.3390/brainsci15030247

**Published:** 2025-02-26

**Authors:** Antonio Del Casale, Darvin Shehu, Maria Camilla Rossi-Espagnet, Clarissa Zocchi, Irene Bilotta, Jan Francesco Arena, Alessandro Alcibiade, Barbara Adriani, Daniela Longo, Carlo Gandolfo, Andrea Romano, Stefano Ferracuti, Alessandro Bozzao, Antonio Napolitano

**Affiliations:** 1 Department of Dynamic and Clinical Psychology and Health Studies, Faculty of Medicine and Psychology, Sapienza University of Rome, 00189 Rome, Italy; janfrancesco.arena@uniroma1.it; 2 Medical Physics Department, Bambino Gesù Children’s Hospital, 00165 Rome, Italy; darvin.shehu@opbg.net; 3 Functional and Interventional Neuroimaging Unit, Bambino Gesù Children’s Hospital, IRCCS, 00165 Rome, Italy; mcamilla.rossi@opbg.net (M.C.R.-E.); daniela.longo@opbg.net (D.L.); carlo.gandolfo@opbg.net (C.G.); antonio.napolitano@opbg.net (A.N.); 4 Psychiatric Diagnosis and Treatment Unit, S. Giovanni Evangelista Hospital, Department of Mental Health, Local Health Authority ASL Roma 5, 00019 Tivoli, Italy; clarissa.zocchi@uniroma1.it; 5 Psychiatric Diagnosis and Treatment Unit, S. Camillo de Lellis Hospital, Department of Mental Health, Local Health Authority ASL Rieti, 02100 Rieti, Italy; irene.bilotta@uniroma1.it; 6 Marina Militare Italiana (Italian Navy), Ministry of Defence, Piazza della Marina, 4, 00196 Rome, Italy; alessandro.alcibiade@hotmail.com; 7 Department of Neuroscience, Mental Health and Sensory Organs (NESMOS), Faculty of Medicine and Psychology, Sapienza University of Rome, 00189 Rome, Italy; barbara.adriani@uniroma1.it (B.A.); andrea.romano@uniroma1.it (A.R.); alessandro.bozzao@uniroma1.it (A.B.); 8 Unit of Neuroradiology, Sant’Andrea University Hospital, 00189 Rome, Italy; 9 Department of Human Neuroscience, Faculty of Medicine and Dentistry, Sapienza University of Rome, 00185 Rome, Italy; stefano.ferracuti@uniroma1.it; 10 Unit of Risk Management, Sant’Andrea University Hospital, 00189 Rome, Italy

**Keywords:** autistic disorder, neuroimaging, neuronal plasticity, prefrontal cortex, morphometric similarity network analysis

## Abstract

**Background**: Autism Spectrum Disorder (ASD) is a neurodevelopmental disorder characterized by persistent difficulties in social interaction, communication, and repetitive behaviors. Neuroimaging studies have revealed structural and functional neural changes in individuals with ASD compared to healthy subjects. **Objectives**: This study aimed to investigate brain network structural connectivity in ASD using Morphometric Similarity Network (MSN) analysis. **Methods**: Data from the Autism Brain Imaging Data Exchange (ABIDE) were analyzed, comprising 597 individuals with ASD and 644 healthy controls. Structural connectivity was assessed using cortical morphometric features. Global and regional network indices, including the density index, node degree, node strength, and clustering coefficients, were evaluated. **Results**: Among the global network indices, when using a threshold value of 0.4, ASD patients compared to HCs showed a lower density (*p* = 0.041) and higher negative clustering (*p* = 0.0051) coefficients. For regional network indices, ASD patients showed a lower bilateral superior frontal cortices degree (left hemisphere: *p* = 0.014; right hemisphere: *p* = 0.0038) and strength (left: *p* = 0.017; right: *p* = 0.018). Additionally, they showed higher negative clustering coefficients in the bilateral superior frontal cortices (left, *p* = 0.0088; right, *p* = 0.0056) and bilateral pars orbitalis (left, *p* = 0.016; right, *p* = 0.0006), as well as lower positive clustering in the bilateral frontal pole (left, *p* = 0.03; right, *p* = 0.044). **Conclusions**: These findings highlight significant alterations in both global and regional brain network organization in ASD, which may contribute to the disorder’s cognitive and behavioral manifestations. Future studies are needed to investigate the pathophysiological mechanisms underlying these structural connectivity changes, to inform the development of more targeted and individualized therapeutic interventions for individuals with ASD.

## 1. Introduction

Autism Spectrum Disorder (ASD) is a neurodevelopmental condition characterized by persistent difficulties in social interaction, communication, and restricted or repetitive behaviors [1]. The global incidence of ASD is estimated to be approximately 0.76%, with variations depending on country and diagnostic criteria. In 2021, an estimated 62 million people worldwide were on the autism spectrum, with a global prevalence of approximately 788 per 100,000 individuals. ASD contributed to 11.5 million disability-adjusted life years (DALYs), with rates highest in high-income regions and lowest in Southeast Asia, East Asia, and Oceania. The burden was evident across all ages, peaking in early childhood and ranking among the top ten causes of non-fatal health burden in individuals under 20 years [2].

ASD is more frequently diagnosed in males than in females, with an estimated ratio of 4:1, and its etiology involves a complex interplay of genetic and environmental factors [3]. ASD can be influenced by genetic, biological, and environmental factors that impact brain development. ASD indeed affects multiple aspects of brain anatomy, making its neuroanatomical correlates inherently challenging to characterize [4].

Neuroimaging studies of ASD have revealed significant structural [5,6,7] and functional [8,9,10] alterations in multiple brain regions, including the prefrontal cortex, amygdala, and cerebellum. Altered connectivity patterns, particularly within the default mode network, have been consistently observed and are thought to underlie social and cognitive impairments in ASD [7,11,12]. Additionally, abnormal white matter integrity, especially in the corpus callosum, has been implicated in the disrupted communication between brain hemispheres often associated with ASD [13,14].

Morphometric Similarity Network (MSN) analysis represents a significant advancement in understanding macroscale cortical organization [15]. This technique assesses the neural structural organization of the brain by evaluating the similarity in morphometric features, such as cortical thickness, surface area, and volume, across different regions. By constructing a network based on these similarities, MSN analysis can shed light on the interconnected structural properties of brain regions. It has been applied to the study of different conditions and diagnoses, including major depression [16,17], responses to childhood trauma [18], psychopathological symptoms in Parkinson’s disease [19], and schizophrenia [20,21,22].

While classical functional connectivity captures dynamic, time-dependent interactions that reveal how brain areas communicate during rest or tasks, MSN provides insight into the brain’s anatomical architecture and the structural relationships between regions. Thus, MSN reflects long-term structural connectivity, whereas functional connectivity captures transient neural interactions. Network metrics such as density, clustering coefficient, degree, and strength can offer insights into the structural and functional connectivity of the brain [20,23]. Given the limited literature on brain morphometric similarity in individuals with ASD, this method is particularly valuable for investigating the neuropathophysiological correlates of the condition. It can reveal structural connectivity alterations that may underlie cognitive and behavioral deficits.

In this study, we hypothesize that global and regional network indices may differ significantly between patients with ASD and healthy control subjects (HCs), reflecting long-term structural connectivity alterations associated with ASD neuropathophysiology. By examining both global and regional network metrics, we aim to gain insights into the structural connectivity patterns associated with ASD neuropathophysiology. Furthermore, the study aims to assess whether network alterations may correlate with the severity of clinical symptoms in ASD. The main objective is to enhance our understanding of ASD’s structural connectivity, which may contribute to the development of targeted interventions.

## 2. Methods

### 2.1. Participants

This study used data from the Autism Brain Imaging Data Exchange (ABIDE) database [24], which aggregates MRI scans from 19 international institutions. For each participant, high-quality 3D T1 MPRAGE (Magnetization Prepared Rapid Gradient Echo Imaging) MRI images, showing no morphological abnormalities, were available for analysis. All data are fully anonymized in accordance with Health Insurance Portability and Accountability guidelines. Details of diagnostic criteria, acquisition, informed consent, and site-specific protocols are available at “http://fcon_1000.projects.nitrc.org/indi/abide/ (accessed on 2 January 2025)”.

### 2.2. Data Processing

Seven morphometric features were derived from the 3D T1 MPRAGE MRI sequences for each participant. The 3D T1 MPRAGE data were preprocessed using FreeSurfer v7, available at “http://surfer.nmr.mgh.harvard.edu (accessed on 2 January 2025)”, through the standard automatic pipeline (i.e., recon-all), which includes skull stripping, removal of non-brain tissue, motion correction, and transformation to Talairach–Tournoux space to generate gray matter (GM) and white matter (WM) segmentation. The inner cortical surface (white surface) was generated by tessellating the GM-WM boundary, while the outer surface (pial surface) was created by expanding the white surface with point-to-point matching.

The calculated morphometric features included the cortical thickness (CT), surface area (SA), gray matter volume (GM), curvature index (CI), folding index (FI), mean curvature (MC), and Gaussian curvature (GC). The CT was measured as the average shortest distance between the estimated gray/white and pial surfaces following the Fischl and Dale approach. The SA was computed as the total area of all vertices within a region. Regional GM volume was the sum of the voxels in a region. The CI represented the maximum intrinsic curvature across all points on the surface. The FI was calculated as the ratio of cortices buried within sulcal folds to those on the visible outer cortex. The MC was computed as the reciprocal of the radius of the inscribed circle for each vertex, with opposite signs for gyri and sulci. GC was the product of the principal curvatures, which were the eigenvalues of the shape operator at a given point. These features were parcellated using an atlas with 34 cortical regions per hemisphere derived from the Desikan cortical atlas.

### 2.3. MSN Analysis

Each morphometric feature vector was normalized using Z-scores across regions to account for value distribution variations between features. Pearson’s correlation analysis was performed on the morphometric feature vector between each paired cortical region, forming a 68 × 68 morphometric similarity matrix (Mi) for each participant. This matrix represented the morphometric similarity (MS) estimated connectivity for each subject, resulting in a weighted matrix with values ±1.

#### 2.3.1. Connectivity Indices

Connectivity indices were computed using the Brain Connectivity Toolbox, available at “https://sites.google.com/site/bctnet/home (accessed on 2 December 2024)”, for each participant’s MS matrices. Four indices were evaluated: two regional (Degree and Strength) and two global (Density and Clustering Coefficient).

- Node Degree: This regional index indicates the number of links of each node. Matrix thresholding was applied to obtain a binary matrix representative of the links. A threshold of 0.4 was selected, as it ensures a balance between sensitivity and stability, given that variation in brain connectivity indices decreases as the threshold increases [25].

The degree of the *i*-th node was computed as the following:

$$D_{i}=\sum_{j=1}^{68}b_{ij}$$

where *b_ij_* is the value at the *i*-th column and *j*-th row of the binarized matrix.

- Node Strength: Unlike the node degree, strength measures the intensity of a node’s connections. Node strength is determined as the sum of weights connected to the node. Positive and negative strengths were calculated as follows:

$${Sp}_{i}=\sum_{j=1}^{68}p_{ij}$$

$${Sn}_{i}=\sum_{j=1}^{68}n_{ij}$$

where *Sp_i_* is the *i*-th node’s positive strength, *p_ij_* is the positive weight in the *ij*-th position, *Sn_i_* is the *i*-th node’s negative strength, and *n_ij_* is the negative weight in the *ij*-th position.

- Density Index: This global index is derived from node degrees and estimated as follows:

$$D=\frac{K}{\frac{\left(N^{2}-N\right)}{2}}$$

where *K* is the number of nonzero weights after thresholding, and *N* is the number of nodes. Density represents the fraction of present links to possible ones, describing the connection density of the brain network graph.

- Clustering Coefficient: This index measures the network’s tendency to form clusters.

$$C_{i}=\frac{1}{k_{i}(k_{i}-1)}\sum_{j,k}{\left({\hat{w}}_{ij}{\hat{w}}_{ik}{\hat{w}}_{jk}\right)}^{1/3}$$

where $k_{i}$ is *i*-th node’s degree, and ${\hat{w}}_{*=\frac{w_{*}}{max(w)}}$ so edge weights are normalized by the maximum weight in the network. Thus, triangles in which each edge has maximum value contribute unity to the sum, while a triangle with just one negligible weight will have a light contribution.

In weighted signed networks, we measured two types of clustering coefficients:

- Positive Clustering Coefficient ($C_{i}^{+}$): Computed by considering only positive edge weights, this measure captures the tendency of nodes to form clusters based on positive relationships.
- Negative Clustering Coefficient ($C_{i}^{-}$): Calculated using only negative edge weights, this coefficient reflects clustering behavior in terms of negative interactions.

#### 2.3.2. Statistical Analysis

We assessed differences in categorical variables using the chi-square test, and for continuous variables, we used Student’s *t*-test with the online tools provided by Social Science Statistics, available at “https://www.socscistatistics.com/ (accessed on 2 December 2024)”. After computing global and local connectivity indices, statistical analysis was performed using the Statistics and Machine Learning Toolbox of MATLAB (version 2020b). A *t*-test was conducted on global indices to determine if ASD brain networks differ globally from control brain networks. Additionally, *t*-tests were applied to local indices for each region to localize the differences between groups. To assess the relationship between ASD severity and connectivity indices, correlation analyses were conducted. The dataset provides scores from the Autism Diagnostic Observation Schedule (ADOS) [26] and its second edition (ADOS-2) [27] as measures of ASD severity. Correlations were therefore computed between ADOS and ADOS-2 scores and each connectivity index across all regions of the atlas.

## 3. Results

The dataset included 1241 participants aged 6 to 64 years, with a mean age of 17.5 years. Participants were divided into two groups: 597 individuals with ASD (524 males, 73 females; mean age = 17.52 years, SD = 9.98) and 644 control subjects (523 males, 121 females; mean age = 17.40 years, SD = 9.31). The chi-square test revealed a significant gender difference between the study groups, with a higher number of females in the control group (χ^2^ = 10.112; *p* = 0.001). Student’s *t* test did not reveal significant age differences between the two groups (t = 0.2269; *p* = 0.8205).

**Figure 1:**
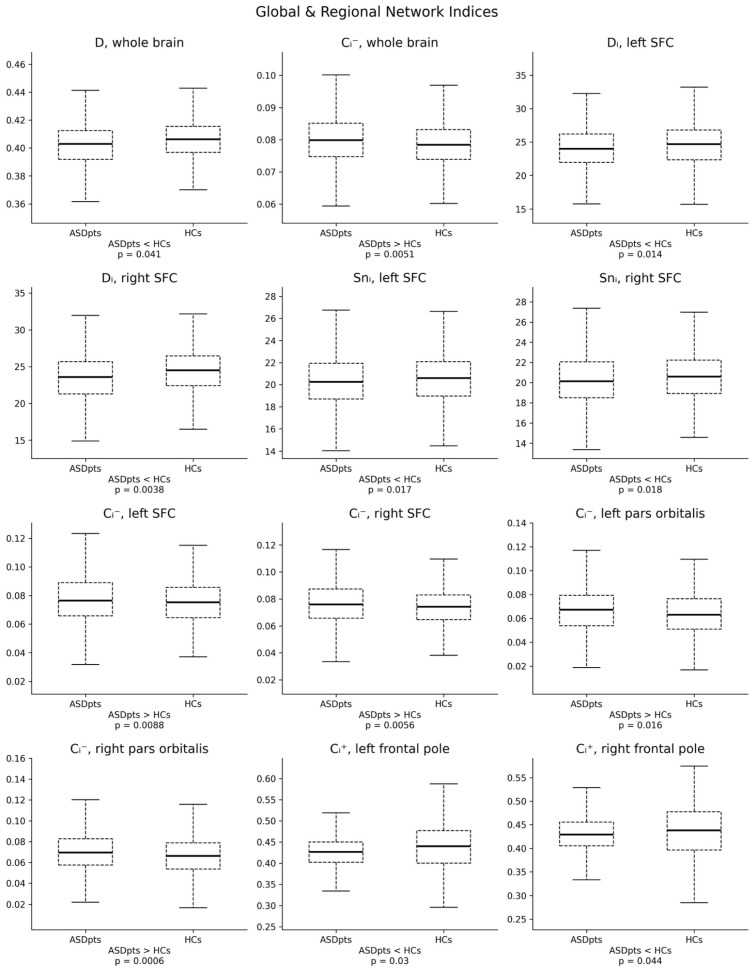
Boxplots representing statistically significant between-groups differences in global and regional brain network indices. Legend: ASDpts = ASD patients; C_i_^−^ = negative clustering coefficient; C_i_^+^ = positive clustering coefficient; D = density index; D_i_ = node degree; HCs = healthy controls; SFC = superior frontal cortex; Sn_i_ = node strength.

Among the global network indices, using a threshold value of 0.4, ASD patients compared to HCs showed lower density (*p* = 0.041) and higher negative clustering (*p* = 0.0051) coefficients. For regional network indices, individuals with ASD demonstrated a lower node degree in the bilateral superior frontal cortices (left hemisphere: *p* = 0.014; right hemisphere: *p* = 0.0038) and reduced node strength in the bilateral superior frontal cortices (left: *p* = 0.017; right: *p* = 0.018). Additionally, ASD patients showed higher negative clustering in the bilateral superior frontal cortices (left, *p* = 0.0088; right, *p* = 0.0056) and bilateral pars orbitalis (left, *p* = 0.016; right, *p* = 0.0006), as well as lower positive clustering coefficients in the bilateral frontal pole (left, *p* = 0.03; right, *p* = 0.044). Correlation analysis between ASD severity and connectivity indices did not reveal any significant associations. We summarize our study findings in Table 1 and Figure 1.

## 4. Discussion

This study found significant differences in brain connectivity between individuals with ASD and healthy controls. Globally, brain networks in ASD are less densely connected and show higher negative clustering, indicating structural dissimilarities and impaired integration between nodes, reflecting a higher antagonistic functioning between nodes, suggesting they are working in opposition or are abnormally disconnected. This suggests abnormal neural relationships, leading to disrupted cohesion and communication within neural circuits. From a pathophysiological point of view, this may reflect pathological segregation, where group of neurons become overly specialized or isolated, reducing network efficiency. Regionally, areas such as the superior frontal cortices showed increased local negative clustering, confirming their abnormal dissimilarity and impaired integration. In contrast, the frontal pole exhibited reduced positive clustering, reflecting weaker structural coherence and disrupted modular organization, which, in turn, suggest lower synergistic functioning between network nodes. These alterations in structural connectivity may contribute to the cognitive and behavioral symptoms observed in ASD.

Focusing on the global indices, we have shown that patients with ASD exhibit a lower global density in their brain networks compared to HCs. The reduced density suggests that their brain networks have fewer actual connections than would be expected given the number of potential connections. From another point of view, the clustering coefficient quantifies the extent to which nodes in a network are inclined to cluster together, with higher values indicating a stronger tendency for nodes to form densely clustered groups. In ASD patients, we observed an elevated negative clustering coefficient. This suggests that ASD patients exhibit a greater prevalence of negatively correlated brain regions that are clustered together, potentially reflecting atypical structural connectivity patterns, and implying a less integrated network structure, which may contribute to the characteristic symptoms of ASD, including difficulties in social communication and interaction. These findings are consistent with the existing evidence of widespread, subtle anatomical and functional differences observed in post-mortem, neuroimaging, and electrophysiological studies of patients with ASD. These differences support various neural developmental hypotheses of ASD [5,28], including those related to overpruning [29], neuroimmunology [30], neural transmission [31], neural signaling [32], genetics [33], and other factors influencing neuronal development and plasticity.

Focusing on regional indices, we identified several changes in network coefficients within the superior frontal cortices of ASD patients. Specifically, we observed a reduced degree index (the number of connections a node maintains within a network) in these regions, indicating diminished connectivity with other brain areas. This reduction in connectivity may impair the integration of information across diverse brain regions, a process essential for higher-order cognitive functions such as executive function and social cognition. The significance of this reduced degree in these critical regions warrants further investigation, as it may illuminate the neural mechanisms underlying the cognitive and behavioral challenges characteristic of ASD.

In addition to a reduced degree, we found that the strength of connections in the superior frontal cortices was also diminished in ASD patients compared to healthy controls. This reduced strength likely reflects a decreased capacity for effective communication between brain regions, potentially contributing to the social and cognitive deficits observed in ASD. The implications of lower strength in specific brain regions highlight the need for targeted interventions aimed at enhancing connectivity and improving outcomes for individuals with ASD.

Furthermore, ASD patients exhibited increased negative clustering in the superior frontal cortices, indicating greater negative interconnectedness among neighboring nodes. Consistent with existing evidence linking cognitive symptoms to alterations in the superior frontal cortex in ASD [34,35], such negative clustering may underlie the distinct cognitive profiles observed in ASD, where certain brain regions may function antagonistically rather than synergistically.

These cognitive anomalies have also been associated with altered mRNA expression of various gamma-aminobutyric acid (GABA) subunits, alongside a global reduction in GABAA receptor protein expression within the superior frontal cortices of individuals with autism [36]. Additional cellular abnormalities identified in this region include dysregulated levels of Bcl-2 and P53, which have been implicated in disrupted apoptotic processes in autism [37]; dysregulation of fragile X mental retardation protein and metabotropic glutamate receptor 5, which are linked to cognitive deficits and seizure disorders in ASD [38]; altered expression of cyclic adenosine monophosphate-specific phosphodiesterase-4 (PDE4) A and B proteins [39]; and increased expression of connexin 43, indicative of abnormal glial–neuronal communication [40]. These and other molecular alterations may contribute to the neurodevelopmental abnormalities and pathophysiology associated with ASD.

Our finding of higher negative clustering in the bilateral pars orbitalis in ASD refers to an increased tendency of neural connections within this brain region to form less tightly knit groups or clusters compared to typical controls. This could lead to altered communication patterns both within and between brain regions, potentially affecting cognitive and behavioral functions. In ASD, such negative clustering may disrupt normal information flow, contributing to the structural and functional abnormalities observed in the brain. This can be linked with evidence connecting differences in the brain-derived neurotrophic factor *val66met* genotype to variations in regional cortical volume and surface area, particularly in the frontal regions, including the pars orbitalis [41]. These findings suggest that altered structural connectivity patterns may be associated with genetic factors influencing neurodevelopment, cerebral morphometry, and connectivity in this area. Other functional changes in this region, particularly in the inferior frontal gyri, such as activations during action observation [42], language processing [43], and tasks involving pragmatic and theory of mind abilities have been linked to difficulties in interpersonal communication [44]. This highlights the role of the inferior frontal regions in both structural abnormalities and functional deficits commonly observed in ASD, particularly in areas related to social cognition and communication.

We also observed lower positive clustering coefficients in the bilateral frontal pole, confirming that dysconnectivity in this region is a key neuropathological correlate of ASD. Several studies have demonstrated functional changes in this area in ASD patients, specifically in relation to executive functioning [45], language processing [46], and hemodynamic patterns [47]. Furthermore, in Brodmann Area 10 (BA10), a significant enrichment of genomic regions associated with immune functions was found among hypomethylated cytosine-phosphate-guanine sites (CpGs), while genes linked to synaptic membrane processes were enriched among hypermethylated CpGs. This suggests that epigenetic mechanisms in BA10 may also contribute to the neuropathophysiology of ASD [48].

In summary, the pars orbitalis exhibited increased negative clustering, and the frontal pole showed a concurrent reduction in positive clustering. These findings underscore the complexity of brain network organization in ASD, suggesting that dysconnectivity in regions involved in language and social processing (pars orbitalis) [49,50] coexists with weaker connectivity in higher-order executive areas (frontal pole) [51,52]. This may reflect a disruption in the interplay between specialized regional processing and global network integration, potentially contributing to ASD-related cognitive and behavioral traits.

Our findings indicate a global reduction in brain network connectivity in ASD, particularly within prefrontal regions, where a decreased density and increased negative clustering suggest a less integrated network structure. The disruptions in network structural connectivity we observed in ASD suggest that individualized treatment approaches may be necessary to address the heterogeneous nature of the disorder. The superior frontal cortices and pars orbitalis, which showed increased local negative clustering in ASD, are implicated in executive function, social cognition, and emotion regulation [53,54,55]. Our findings support the potential for targeted interventions, such as neurofeedback training [56], transcranial magnetic stimulation (TMS) [57,58,59], and other brain stimulation techniques [60], to modulate connectivity patterns in these regions. Understanding region-specific connectivity alterations can help optimize stimulation protocols to achieve maximal therapeutic effects.

Additionally, interventions focusing on cognitive and behavioral training could be tailored to an individual’s unique neuroanatomical profile. For example, cognitive training programs that engage the frontal lobe in problem-solving and perspective-taking tasks may be particularly beneficial for individuals with ASD who exhibit altered connectivity in the frontal pole. Combining neuroimaging assessments with behavioral interventions could provide a more comprehensive approach to treatment planning.

Altered structural connectivity in ASD may also have implications for pharmacological research. Current pharmacological treatments for ASD primarily target symptoms such as irritability and repetitive behaviors rather than core social and cognitive deficits [61]. However, emerging research suggests that connectivity alterations and cortical excitation/inhibition imbalances in ASD may be linked to dysregulated neurotransmitter systems, including GABA and glutamate signaling [62,63]. The increased clustering in the superior frontal cortices and pars orbitalis observed in this study aligns with previous findings of abnormal GABAergic signaling in these regions [64,65]. This raises the possibility that pharmacological interventions targeting the inhibitory–excitatory balance, such as GABA modulators or glutamatergic agents [66,67], could be explored for their potential to restore network connectivity and improve cognitive functioning in ASD.

This study has several strengths that enhance the robustness and significance of its findings. One of the primary strengths is the application of MSN analysis to investigate structural connectivity alterations in individuals with ASD. Unlike traditional neuroimaging approaches, MSN provides a network-based framework that captures macroscale cortical organization by integrating multiple morphometric features. This method relies solely on structural images, which are easier to acquire and more widely available than other types of MRI acquisition and allows for a more comprehensive characterization of brain network architecture and its deviations in ASD, offering insights that extend beyond localized volumetric or thickness differences observed in previous studies.

Another strength of this study is the large and diverse sample size derived from the ABIDE dataset. By incorporating MRI data from multiple international sites, this study benefits from a broad representation of ASD-related variability, increasing the generalizability of the findings. While multi-site datasets inherently introduce scanner-related variability, the use of standardized preprocessing pipelines, such as FreeSurfer-based cortical parcellation and feature extraction, ensures consistency in data processing across institutions. The ability to detect significant global and regional network differences despite potential site-related noise further supports the robustness of MSN as a structural connectivity analysis tool.

Finally, this study lays the groundwork for future translational applications of MSN in ASD. By demonstrating its potential to characterize altered structural connectivity patterns, these findings support further investigations into the clinical utility of MSN-derived biomarkers for diagnosis, patient stratification, and personalized interventions. Integrating MSN analysis with functional connectivity and genetic studies may provide a more holistic understanding of ASD neuropathophysiology and guide the development of targeted therapeutic strategies.

The primary limitation of this study is the reliance on data from the ABIDE, which aggregates MRI scans from multiple international institutions, potentially introducing variability in imaging protocols and scanner settings. While this heterogeneity poses a challenge, it also enhances the generalizability of findings by encompassing a diverse representation of ASD-related brain alterations across different populations and scanning conditions. To mitigate the impact of inter-site variability, we employed a standardized preprocessing pipeline using FreeSurfer v7, which ensures consistent cortical morphometric feature extraction across all datasets. This pipeline includes skull stripping, motion correction, intensity normalization, and cortical parcellation based on the Desikan-Killiany atlas. Moreover, while site-related variability may introduce some degree of noise, large-scale, multi-center datasets such as the ABIDE have been shown to produce replicable and biologically meaningful findings [68,69,70]. Our results align with prior research on ASD-related connectivity alterations, reinforcing their validity despite potential methodological heterogeneity. Future studies may further address this issue by incorporating harmonization techniques, such as deep learning approaches [71], to reduce site-specific differences while preserving true biological variability. Another potential limitation is the gender imbalance between the ASD and control groups, which may affect the generalizability of the findings, particularly given the known differences in ASD presentation between males and females. However, this imbalance reflects the greater prevalence of ASD in males in the general population – a common challenge in this field.

The clinical translation of our findings will require further validation in independent cohorts and across different developmental stages of ASD. Longitudinal studies are particularly needed to investigate how structural connectivity evolves over time and whether early alterations predict later cognitive and behavioral outcomes. Additionally, integrating MSN analysis with genetic and molecular data may help clarify the underlying pathophysiological mechanisms driving connectivity disruptions in ASD. Advances in artificial intelligence and deep learning algorithms also present opportunities to develop automated neuroimaging pipelines that can be implemented in clinical settings to aid in ASD diagnosis and treatment monitoring.

Overall, the findings of this study contribute to the growing body of evidence supporting the role of disrupted brain network organization in ASD, underlying significant changes in brain structural connectivity. By leveraging structural connectivity analyses in clinical and translational research, future efforts may move closer to developing more precise diagnostic tools and individualized interventions that target the core neuropathophysiological features of ASD.

## 5. Conclusions

In conclusion, this study reveals a complex reorganization of brain networks in ASD patients, characterized by global reductions in network density alongside region-specific connectivity variations as compared to the healthy state. Notably, alterations in regions such as the superior frontal cortices, pars orbitalis, and frontal pole indicate that disruptions in structural connectivity within areas critical for social cognition, thought, executive function, and emotional regulation may underlie the cognitive and behavioral manifestations of ASD. The observed patterns of dysconnectivity suggest complex reconfigurations of global brain networks, highlighting the diverse ways in which ASD affects the brain architecture.

These findings highlight the central role of structural connectivity alterations in the ASD clinical presentation. By elucidating the macrostructural organization of the brain in ASD, this research could pave the way for future studies investigating the pathophysiological mechanisms behind these changes, which could ultimately inform the development of more targeted and individualized therapeutic interventions.

## Figures and Tables

**Table 1:** Statistically significant changes in global and regional network indices in patients with ASD compared to HCs.

Measure, Brain Area	ASDpts Mean (SD)	HCs Mean (SD)	*p*	Findings in ASDpts
*D*, whole brain	0.4027 (0.0157)	0.405 (0.0144)	0.041	ASDpts < HCs Lower number of links of each node
*C_i_^−^*, whole brain	0.0796 (0.0079)	0.0784 (0.007)	0.0051	ASDpts > HCs Higher antagonistic functioning
*D_i_*, left SFC	24.1106 (3.3650)	24.5590 (3.0273)	0.014	ASDpts < HCs Lower connection density
*D_i_*, right SFC	23.9045 (3.4304)	24.4363 (3.0308)	0.0038	ASDpts < HCs Lower connection density
*Sn_i_*, left SFC	20.3458 (2.4928)	20.6695 (2.2609)	0.017	ASDpts < HCs Weaker connections
*Sn_i_*, right SFC	20.2903 (2.5485)	20.616 (2.2806)	0.018	ASDpts < HCs Weaker connections
*C_i_^−^*, left SFC	0.0771 (0.0164)	0.0747 (0.015)	0.0088	ASDpts > HCs Higher antagonistic functioning
*C_i_^−^*, right SFC	0.0763 (0.0172)	0.0738 (0.0149)	0.0056	ASDpts > HCs Higher antagonistic functioning
*C_i_^−^*, left pars orbitalis	0.0671 (0.0194)	0.0645 (0.0191)	0.016	ASDpts > HCs Higher antagonistic functioning
*C_i_^−^*, right pars orbitalis	0.0698 (0.02)	0.066 (0.0193)	0.0006	ASDpts > HCs Higher antagonistic functioning
*C_i_^+^*, left frontal pole	0.4281 (0.0363)	0.4356 (0.0565)	0.03	ASDpts < HCs Lower synergistic functioning
*C_i_^+^*, right frontal pole	0.4285 (0.0367)	0.4354 (0.0575)	0.044	ASDpts < HCs Lower synergistic functioning

Abbreviations: ASDpts, ASD patients; HCs, healthy controls; *D*, density index; *C****_i_****^−^*, negative clustering coefficient; *C****_i_****^+^*, positive clustering coefficient; *D****_i_***, node degree; *Sn****_i_***, node strength; SFC, superior frontal cortex.

## Data Availability

The original data presented in this study are openly available in the ABIDE (Autism Brain Imaging Data Exchange) at “https://fcon_1000.projects.nitrc.org/indi/abide/ (accessed on 2 December 2024)”.
